# Anti-Cancer Vaccine for HPV-Associated Neoplasms: Focus on a Therapeutic HPV Vaccine Based on a Novel Tumor Antigen Delivery Method Using Endogenously Engineered Exosomes

**DOI:** 10.3390/cancers11020138

**Published:** 2019-01-24

**Authors:** Paola Di Bonito, Luisa Accardi, Luisa Galati, Flavia Ferrantelli, Maurizio Federico

**Affiliations:** 1 Department of Infectious Diseases, Viral Hepatitis, Oncoviruses and Retroviruses (EVOR) unit, Istituto Superiore di Sanità, Viale Regina Elena 299, 00161 Rome, Italy; luisa.accardi@iss.it (L.A.); luisagalat@gmail.com (L.G.); 2 National Center for Global Health, Istituto Superiore di Sanità, Viale Regina Elena 299, 00161 Rome, Italy; flavia.ferrantelli@iss.it (F.F.); maurizio.federico@iss.it (M.F.)

**Keywords:** cancer immunotherapy, HPV16, therapeutic vaccines, extracellular vesicles, exosomes, cytotoxic T lymphocytes

## Abstract

Some human papillomavirus (HPV) genotypes are universally recognized as major etiological agents not only of ano-genital tumors but also of head and neck cancers, which show increasing incidence. The evaluation of current and future therapeutic approaches against HPV-induced tumors is a global health priority, despite an effective prophylactic vaccine against 7 of the 12 genotypes involved in the etiology of tumors being currently available. In this review, we present the main anti-HPV therapeutic approaches in clinical experimentation, with a focus on a novel tumor antigen delivery method using engineered exosomes, that we recently developed. Our system allows the induction of an efficient unrestricted cytotoxic T lymphocyte (CTL) immune response against the HPV16-E7 tumor-associated antigen, with the formation of endogenously engineered exosomes, i.e., nanovesicles spontaneously released by all cell types. Immunogenic exosomes are uploaded with HPV16-E7 due to the fusion with a unique exosome-anchoring protein referred to as Nef^mut^. Intramuscular injection of a DNA vector expressing the fusion protein generates exosomes sufficiently immunogenic to elicit a potent anti-16E7 CTL immune response. The approach is described here and the advantages over other existing methodologies are reported.

## 1. Introduction

Despite the significant progress obtained in the past decades in the field of tumor therapy, robust anticancer therapies able to induce effective antitumor responses, namely to clear tumor cells while avoiding adverse effects, are not yet available. Combination therapies are generally considered the most effective way to counteract tumor pathologies [1]. Interest in anticancer immunotherapies has grown explosively over the last 5–10 years mainly due to approval of new clinical protocols based on the use of monoclonal antibodies, referred to as immune check-point blockers (ICBs) [2], which foster pre-existing immune response against both tumor-associated antigens (TAAs) and neo-antigens. The success of immunotherapies based on ICBs definitely proves that cancer can be treated by manipulating the immune system. However, this strategy suffers from some limitations, including intrinsic/acquired resistance and the development of hyper-immune activation that may be associated with immune-related adverse events affecting several organs, including skin, gut, heart, lungs, and bone [3].

Two types of cancer immunotherapy have emerged as the most promising so far: (i) T cell-based cancer immunotherapy, including active vaccination and adoptive cell transfer and (ii) immune modulation through monoclonal antibodies based on an immune checkpoint blockade [4,5].

Cancer cells express a multitude of new antigens due to the inherent genetic instability typical of malignant transformation and/or to the expression of cancer etiological agents, as in the case of malignancies associated with the human papillomavirus (HPV) and the hepatitis B virus. In non-virus-induced cancers, the transformed cells can produce antigens to which the host is tolerant (tumor-associated self-antigens), as in the case of antigens either expressed during fetal development (oncofetal antigens), e.g., Carcinoembryonic antigen (CEA), or overexpressed in specific tissues, e.g., human epidermal growth factor receptor 2 (HER2) in mammary glands. Furthermore, cancer cells can express antigens to which the host does not develop tolerance, however, the immune response is not sufficiently effective to counteract the growth of cancer cells. Such antigens include the so-called tumor-specific neo-antigens as well as antigens normally produced in immune-privileged tissues, such as cancer-testis antigens (CTAs), which in adults are restricted to male germ cells. Hence, establishing a method for inducing an adaptive immune response against both tolerogenic and non-tolerogenic TAAs could provide the basis for designing innovative therapeutic antitumor approaches.

The immune system can be manipulated in different ways according the antitumor mechanisms that are to be exploited. In most cases, the immune system is primed and boosted via antigen-presenting cells (APCs), T-cells, or innate cells stimulation, to reduce immunosuppression in the tumor environment by regulating inhibitory pathways and enhancing adaptive and/or innate immunity. Immunotherapies can be classified as active or passive based on how they interact with the immune system [6]. Active immunotherapies stimulate the immune system by presenting antigens to elicit a specific immune response, while passive immunotherapies attack the tumor directly, without directly engaging the patient’s immune system to target a specific antigen.

In this context, cancer vaccines can be used to prime or boost the immune system by generating or amplifying tumor antigen-specific immune responses against proteins differentially expressed by tumor cells. Several different approaches include synthetic tumor peptides (either allogenic or autologous), dendritic cell (DC)-based vaccines, and genetic vaccines (DNA/RNA/virus/bacterial). Overall, vaccine strategies aiming at inducing a pool of tumor-specific CD8^+^ T cells as well to reactivate pre-existing anergic tumor-specific CD8^+^ T cells, would be of great benefit for the development of new, safer, and more effective anticancer strategies [7].

In addition to impacting public health and science, anti-cancer vaccines are also moving huge economic interests. Together with cell therapies, they represent the largest immuno-oncology drugs (IO) classes. In the last year, with the extraordinary increase in the number of active agents, drug targets, and clinical-stage organizations, the most active big pharma in this field seem to be Bristol-Myers Squibb, Celgene, and Novartis, with 4 academic centers within the 15 top pipelines [8].

The need for cancer vaccines prompted intense research activity and hundreds of clinical trials are under way. However, the success rate is very low for the time being. Until 2017, only Provenge (sipuleucel-T, a dendritic cell vaccine) achieved the Phase III trial as a primary endpoint that led to regulatory approval. 

In this review, we described the most advanced therapeutic approaches against HPV-associated tumors and compared their performance to that of a novel pre-clinical vaccine based on the in vivo engineering of exosomes released spontaneously by muscle cells.

## 2. HPV-Related Tumors

Human papillomaviruses (HPVs) are small icosahedral viruses of approximately 55 nanometers with a circular double strand DNA genome of about 8000 nucleotides. The genome has three functional regions: The long control region (LCR), the early region coding for the E6, E7, E1, E2, E4, E5 non-structural proteins, and the late region encoding the L2 and L1 structural proteins. The oncogenic potential of the HPVs resides in the activity of the E6, E7, and E5 proteins. In some HPV genotypes, these proteins are oncogenic both in vitro and in vivo. During infection, E6 and E7 are responsible for the breakdown of cell cycle control through the alteration of the activity of tumor suppressors p53 and pRB, respectively. E5 is an integral membrane protein that cooperates with E6 and E7 in cell transformation and epithelial hyperplasia [9,10]. HPV infects basal keratinocytes of skin and mucosa, and replicates only during the process of differentiation of infected cells, inducing the hyper-proliferation of stratified squamous tissues. For this peculiar characteristic, in vitro cellular systems that allow HPV replication are difficult to develop and unavailable to date. The lack of proper cellular and animal models has delayed the comprehension of the oncogenic mechanisms underlying the HPV pathogenesis [10].

Infections with HPVs belonging to the alfa genus are the sexually transmitted diseases most commonly diagnosed in many countries [11,12]. Molecular and epidemiological studies have clearly demonstrated that several genotypes of the alpha genus, referred to as high-risk (HR) HPVs, have oncogenic potential for humans and are classified in the group I of carcinogens by the International Agency for Research on Cancer (IARC). Worldwide, the HR HPV16 and HPV18 account for 71% of cervical cancer (CC) cases. HPV16 and 18 also cause most anal, vulvar, vaginal, and penile carcinomas. Recently, HPV16 and 18 have been confirmed as causative agents of oropharyngeal tumors, in particular tonsillar carcinomas and base tongue carcinomas [11,12].

Overall, 610,000 cancers per year are attributable to HPV infection globally and, in particular, the CC has an annual incidence of 528,000 new cases with over 266,000 deaths [13]. This cancer is among the most frequent cause of death for women in fertile age living in low-income countries, where more than 80% of the global CC cases occur [13]. Conversely, in high-income countries cervical cancer is decreasing thanks to screening campaigns and prophylactic vaccination, while oropharyngeal tumors caused by HPVs are increasing. Globally, 585,000 new cases of head and neck cancers are diagnosed annually, 90% of which are squamous cell tumors. The most recent estimates report that about 38,000 cases of head and neck cancers are attributable to HPVs [12], with a constant increase in the number of cases. In the Unites States, the significant increase in the incidence of HPV-mediated oropharyngeal cancer has been strongly highlighted [14]. Studies of HPV prevalence established that HPV16 and 18 are responsible for 90% of the HPV-mediated oropharyngeal cancers [15,16].

It has been established that about 5% of all tumors are caused by HPVs worldwide; moreover, numerous studies suggest the involvement of the alpha HR-HPVs in additional types of tumors [17], including those affecting the esophagus [18], the lung [19], prostate [20,21], endometrium [22], breast [23], colorectal [24,25], and urinary tract [26]. However, epidemiological studies are still needed to establish whether the virus is the etiologic tumor agent or just an infecting passenger. On the other hand, the spread of HPVs should not be surprising, since studies on water environments demonstrated the presence of HR-HPVs in urban wastewater, river water, swimming pool waters, and spas [27,28]. For these reasons, HPVs have been included among the excreted pathogens potentially dangerous for human health by the Global Water Pathogen Project (http://www.waterpathogens.org/) [29].

## 3. Current Anti-HPV Therapeutic Strategies

Thus far, numerous HPV therapeutic vaccine formulations have been developed, most of which induce an adaptive immune response against the E6 and E7 oncoproteins of HPV16 and/or HPV18. Therapeutic approaches for HPV-associated malignancies involve the use of live bacterial or viral vectors, peptides, recombinant proteins, and nucleic acids. The E6 and E7 proteins have been fused either to each other to form a unique protein, or to different sorting signals to enhance antigen presentation by professional APCs. They have been detoxified by mutagenesis of the p53 and pRB binding sites and their genes have been optimized to obtain higher expression improving the immunogenicity in the host. Several vaccines have reached the clinical trial, where some did not pass the phase 1 clinical trial as a consequence of the low cytotoxic T lymphocyte (CTL)-specific immune response induced in healthy subjects, and others showed promising results for the treatment of CC and CIN1/CIN2 cervical lesions in terms of both safety and efficacy [30]. Table 1 shows some of the therapeutic vaccines against the HPV malignancies in clinical trials as reported by clinicaltrial.gov [31]. Currently, most of the therapeutic HPV vaccines undergoing clinical evaluation are DNA vaccines administered in combination with: (i) conventional chemotherapeutic drugs and radiotherapy; (ii) immune stimulatory molecules such as cytokines and adjuvants; (iii) monoclonal antibodies blocking immune checkpoints blockade and/or angiogenesis. The main DNA-based anti-HPV therapeutic approaches developed over time [32] are the following: eukaryotic vectors expressing HPV16 E7 and E6 fused to different sorting signals (either from LAMP-1, HSP70, *Mycobacterium bovis* HSP65, or Herpes Simplex Virus (HSV) VP22 protein) to improve antigen presentation by the professional APCs [33]; eukaryotic vectors expressing HPV16-E6 and E7 fused to the calreticulin sorting signal [34]; a Cytomegalovirus (CMV)-promoted eukaryotic vector expressing a codon-optimized HPV16-E6 sequence [35]; a pVAX vector expressing a consensus HPV18-E6/E7 sequence obtained from the multiple alignment of 12 HPV-E6 and -E7 gene of HPV18 variants isolated in different countries (p18ConE6E7) [36]; a pVAX vector expressing a consensus HPV16-E6/E7 sequence deduced from a multiple sequence alignment of E6 and E7 genes of the prevalent HPV16 variants isolated in different countries (p16ConE6E7) [37]; GX-188E, i.e., the CMV-promoted pGX10 vector expressing a shuffled, codon-optimized open reading frame consisting of fragments of both the E6 and E7 of HPV16 and HPV18, engineered at the N-terminus with both the signal sequence of the tissue-type plasminogen activator (tPA) to target the secretory pathway and the Fms-like tyrosine kinase-3 ligand (Flt3L) to promote the antigen presentation [38].

## 4. Exosomes in Cancer Immunotherapy

The field of exosome-based cancer therapeutics was launched two decades ago, with two seminal publications highlighting the potential of dendritic cell- and tumor-derived exosomes in cancer immunotherapy [58,59]. Currently, exosomes are considered as potential new biopharmaceuticals and vaccines for the treatment and prevention of several diseases, and their development is very active.

**Figure 1:**
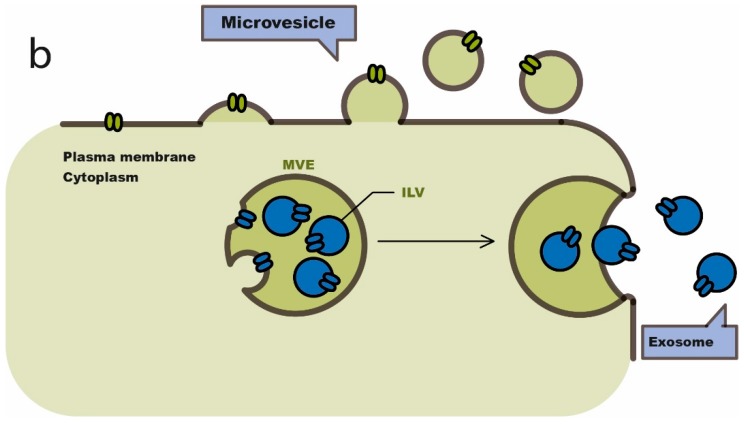
Scheme of the biogenesis of exosomes and microvesicles. Modified from Van Niel, 2018 (Concession Nature Review) [60]. ILV: intraluminal vesicle; MVE: multivesicular endosomes.

Extracellular vesicles (EVs) comprise a heterogeneous population of membrane vesicles of various origins. Their size may typically vary between 50 and 500 nm. Over the past two decades, extracellular vesicles were named based on their origin (cell type), size, morphology, and cargo content, but they are currently classified in two distinct major classes: exosomes (50–150 nm) and microvesicles (Mvs, 100–500 nm), as shown in Figure 1. The inward invagination of endosomal membranes gives rise to the formation of intraluminal vesicles (ILVs) belonging to multivesicular bodies (MVBs). MVBs can be either degraded by lysosomes or fused to plasma membrane thus originating exosomes which release their contents in the extra-cellular milieu [60].

The exosome immunogenicity basically relates to the amount and quality of associated antigens. *Trans*-membrane proteins such as Mart-1, gp100, TRP-1, Her2/neu, and CEA, represent TAAs spontaneously associating to exosomes, that can activate specific anti-tumor T cell immunity [61,62]. Exosomes deriving from APCs expose Major Histocompatibility Complex (MHC) Class I- and II-peptide complexes which can be presented to T lymphocytes either directly, thus increasing T-cell activation due to co-stimulatory molecules incorporated in the membrane, or indirectly upon internalization in dendritic cells (DCs). In this case, the rejection of established tumors can occur due to the exosomes bearing Class I MHC-tumor peptide complexes. Nevertheless, it should be noted that cancer patients can even show production of exosomes and no anti-tumor response, probably owing to immunosurveillance evasion [63].

TAA-associating exosomes have been tested as cell-free vaccines in a number of clinical trials on late-stage tumor patients [64,65,66]. For instance, DC exosomes carrying melanoma-associated antigen (MAGE)-A3 peptides were used for vaccination of patients bearing MAGE-A3^+^ advanced melanomas. The vaccination of 15 subjects with melanoma generated an unambiguous response in one patient, a minor response in another one, and the stabilization of the disease associated with tumor regression in two other patients. An enhanced effector function of natural killer (NK) cells was shown in eight patients [66].

In general, clinical studies have demonstrated a good tolerability of exosomes as cell-free vaccines. However, therapeutic efficacy appeared quite limited. Recent research has highlighted the versatility and intriguing opportunities offered by exosomes to address different unmet clinical needs. Emerging issues concern the functionalization of exosomes that could impact on their efficacy, safety, and specificity in order to improve their clinical development [63].

Current approaches are based on the engineering of EVs (i) isolated from ex vivo cell cultures, (ii) purified from biological fluids (plasma, serum, urine, and saliva), and (iii) produced in vitro from cultured cells. Although exosomes are in principle viable biological entities for therapeutic approaches, several challenges, both scientific and logistical, remain before their clinical potential could be fully realized.

## 5. The Nef^mut^-Based Technology for the Induction of Anti-Tumor CTL Immune Response by Endogenous Engineered Exosomes

The generation of a strong cytotoxic T lymphocyte (CTL) immune response is universally considered a critical end-point for any anti-tumor therapeutic vaccine strategy. We developed an innovative strategy based on the induction of an effective anti-HPV-E7 CTL immunity. The approach relies on in vivo delivery of a DNA vector leading to the generation of engineered exosomes expressing a TAA so that a potent and specific CTL immune response is elicited in the receiving organism. The idea was based on two key findings. The first is the identification of a mutant of a Human Immunodeficiency Virus 1(HIV-1) Nef protein (Nef^mut^) acting as an exosome-anchoring protein. HIV-1 Nef is a 27 kilodalton protein without enzymatic activities [67]. Nef is anchored to membranes through its myristoylated N-terminus and a stretch of basic amino acids located in alpha helix loop 1. Nef is associated with lipid raft microdomains and found in both exosomes and HIV viral particles. Nef^mut^ is a functionally defective protein mutant lacking the Nef functions typically associated with HIV pathogenesis, as shown in Table 2.

Nef^mut^ shows a remarkable ability to incorporate into exosomes and, more in general, into extracellular vesicles (EVs), an ability from 50 to 100 folds higher compared to that of the wild type isoform [68]. Nef^mut^ fused to antigens of choice still retains the abilities of exosome anchoring and incorporation of high amounts of antigens, which remain inside the EVs protected from external neutralization and/or degradation factors [70]. The second key finding concerns the possibility of generating antigen-incorporating EVs in vivo, by intramuscular injection of a DNA vector expressing the antigen of choice fused to Nef^mut^. The in vivo engineered exosomes are immunogenic because they can induce a CTL response both specific and potent.

**Figure 2:**
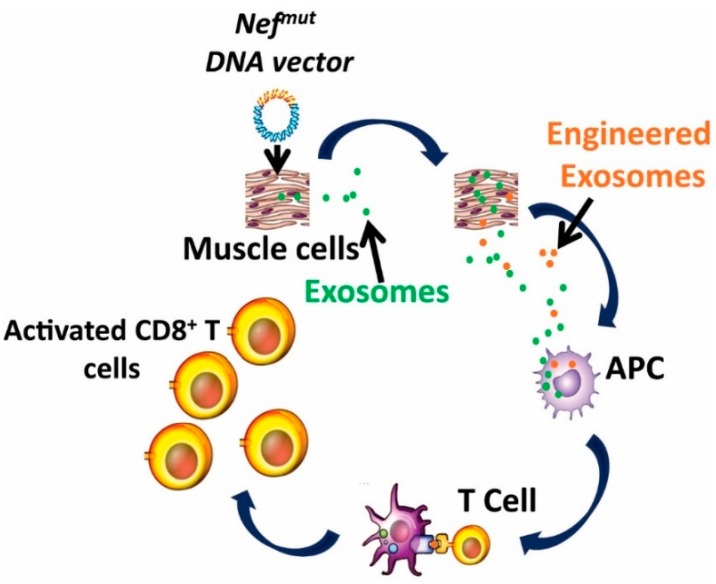
Rationale of the Nef^mut^-based CTL vaccine platform (concession IJN: December 2017) [77]. APC: antigen-presenting cell.

Our platform technology, as shown in Figure 2, is based on:

- The high level of incorporation of the Nefmut protein into EVs [68];
- The Nefmut ability to act as an exosome-anchoring element upon fusion with heterologous proteins [67];
- The experimental evidence that Nefmut-based exosomes loaded with an antigen of choice produced in vitro, induce a strong CTL activity when inoculated in mice [76];
- The possibility to generate recombinant EVs carrying the fusion product of Nefmut—with an antigen of choice in vivo, through intra muscular (i.m.) injection in mice of a DNA vectors coding for the fused genes [76];
- The therapeutic antitumor effect induced by endogenously engineered EVs incorporating HPV-E7 fused with Nefmut [77].

## 6. The Nef^mut^-Based Technology for the Therapy of HPV-Associated Tumors

Recently, it was demonstrated that exosomes engineered in vitro to upload high amounts of HPV-E7 fused to Nef^mut^, elicited an efficient anti-E7 CTL immune response when injected in mice [77]. In view of a potential clinical application of this system, and to overcome the difficulties of EV isolation and purification [67], it was investigated if intramuscular immunization of mice with the DNA vector expressing the Nef^mut^/E7 fusion protein could provide the animals with a continuous source of endogenously engineered EVs able to elicit an effective E7-specific immune response. First, it was demonstrated that injection of a Nef^mut^/green fluorescent protein (GFP)-expressing vector caused the release of fluorescent exosomes detectable in the plasma of inoculated mice. Then, it was shown that mice inoculated with a plasmid expressing Nef^mut^/E7 developed a CD8^+^ T cell immune response against both Nef^mut^ and E7.

Furthermore, E7-loaded EVs were isolated from the plasma of mice immunized with the Nef^mut^/E7 DNA plasmid and used as immunogens in a different group of mice. An E7-specific CTL immune response was revealed in this group of animals, demonstrating that the immunogenicity was really induced by the transfer of recombinant EVs. The therapeutic efficacy of this approach was confirmed by the potent anti-tumor activity generated in the mice injected with the Nef^mut^/E7 plasmid after implantation of TC-1 tumors. Such E7-specific CTL activity was associated with the reduction of the TC-1 tumor burden in mice, and in contrast, no CD8^+^ T cell responses were detected in mice immunized with plasmids expressing only the Nef Wild-Type (wt) isoform, nor in those expressing E7 alone [77].

## 7. Other Applications of the Nef^mut^-Based Exosome Technology

The proof-of-principle of the immunogenicity of in vivo engineered exosomes, obtained using the fusion Nef^mut^-HPV-E7, was then extended to other viral protein antigens involved in diseases for which a CTL-vaccine would benefit. The VP24, VP40, and NP proteins of the Zaire Ebola virus, the NP protein of the influenza virus, the NP of the Crimean-Congo hemorrhagic fever virus, and the NS3 proteins of the West Nile virus and hepatitis C virus (HCV) [78], were fused to Nef^mut^ for expression in exosomes. All the fusion proteins were stable and expressed at a level comparable to Nef^mut^. When the respective DNA plasmids were used in experiments of mice immunization, significant amounts of antigen-specific CD8^+^ cells with cytotoxic activity able to kill peptide-loaded and/or antigen-expressing syngeneic cells, were produced [78]. These data demonstrate that this innovative CTL vaccine platform is efficacious, flexible, and applicable to a wide range of viral diseases. 

We also investigated two additional key issues: (i) whether the immunogenic stimulus induced by the in vivo engineered EVs can break immune tolerance and (ii) the effectiveness of in vivo engineered EVs when applied to a human system [79]. As a model of immune tolerance, we used transgenic mice for the expression of activated rat HER2/neu which spontaneously develop adenocarcinomas in all mammary glands. This is a well characterized mouse model system developing a very aggressive disease and has been used in many investigations including DNA vaccination studies (for an example, see [80]). We showed that when HER2/neu transgenic mice were injected with a DNA vector expressing the fusion product of Nef^mut^ with the extra-cellular domain of HER2/neu, antigen-specific CD8^+^ T lymphocytes became readily detectable. This immune response was associated with a HER2-directed CTL activity and a significant delay in tumor development. These data indicate that the CTL vaccine platform has the potential to be developed as a new immunotherapeutic strategy also against tumors expressing self-antigens, i.e., products highly expressed in oncologic lesions but tolerated by the immune system.

In addition, data from cross-priming experiments where human DCs isolated from co-cultures with DNA-transfected human primary muscle cells were used as APCs, supported the idea that the Nef^mut^-based vaccine platform works also in the human system [70].

## 8. Pros and Cons of the Nef^mut^/E7 Based DNA Vaccines

In the classic DNA vaccine designs, the antigens expressed by the recipient muscle cells upon i.m. DNA injection can be secreted, thereby generating a humoral adaptive immune response. Muscle cells are not professional APCs and do not express co-stimulatory molecules. Hence, the CD8^+^ T immune response detected after i.m. injection of DNA vaccines can basically be a consequence of the capture and expression of the DNA molecules by professional and semi-professional APCs (e.g., DCs, endothelial cells) embedded in the muscle tissue. In this case, the degradative products of the DNA vector-expressed antigens can readily associate with MHC Class I molecules which, in the presence of co-stimulatory molecules, can elicit antigen-specific CD8^+^ T lymphocytes. However, naked DNA does not spread from cell to cell in vivo and APCs do not readily take up expressed antigens, nor do they activate satisfactory immune responses. The delivery of DNA vaccines in DCs can be achieved most effectively through subcutaneous/intradermal injections, for instance through gold particles/gene gun. In any case, DNA-based human cancer vaccines still suffer from low immunogenicity, which hampers the desired clinical success. Thus, effective strategies enhancing the DNA vaccine potency need to be developed. All DNA vaccines under development are at an early stage and none has reached the market so far. To overcome such defects, ex vivo treatments of DCs with RNA or vectored vaccines are currently under investigation.

On the other hand, our CTL vaccine platform combines the remarkable benefits of efficient cross-presentation of EV-associated antigens and the consequent specific induction of a potent CD8^+^ T immune response, with several advantages typical of DNA vaccines, including: (i) simple and flexible design, so that a wide range of antigens and immunomodulatory molecules can be expressed; (ii) unrestricted MHC Class I use; (iii) no unsafe infectious agents involved in the preparation of immunogens and, consequently, no adverse clinical effect or toxicity and no production of anti-DNA antibodies, which allows repeated administrations; (iv) great heat stability and ease of storage and transport without the need for a cold chain; and (v) cost effectiveness. Immunogens can be developed quickly and easily once the antigen has been identified. The production can be very rapid, reproducible, and perfectly suitable for large-scale production and administration.

Differently from other therapeutic DNA vaccines, in the Nef^mut^-based biotechnology platform based on the injection of a eukaryotic DNA-vector, the expressed antigen is incorporated into the EVs spontaneously released by DNA-transfected muscle cells. These EVs are supposed to circulate also in body compartments distal from the injection site, thereby encountering professional APCs. By ingesting engineered EVs, APCs cross-present the associated antigen, hence inducing antigen-specific CTLs.

Notably, no reproducible size constraints were found during the generation of engineered exosomes. In addition, membrane molecules for the EV recognition of specific cell targets can be co-expressed. In Table 3, the main advantages of the Nef^mut^-based CTL vaccine platform are summarized.

As far as concerns of humoral immunity, the Nef^mut^-based approach seems to be unable to induce humoral immune activation, since no production of antibodies against Nef^mut^ and/or the fused antigens was detected in any of our experiments. Additional current limitations are represented by a number of still unclear in vivo mechanistic aspects (e.g., biodistribution and pharmacokinetic of engineered EVs) which, however, are expected to be clarified by ongoing investigations.

In view of a clinical use of the Nef^mut^-based anti-HPV strategy, the evaluation of its potency, in terms of activation of specific CD8^+^ T lymphocytes, was performed by comparison with other DNA-based HPV therapeutic vaccines. Table 4 shows the results of IFN-γ ELISPOT assays performed in different preclinical studies, where the percentage of CD8^+^ activation was calculated considering that the CD8+ T lymphocyte sub-population represents about 30% of total splenic lymphocytes, which, in turn, are around 30% of total splenocytes. Interestingly, by restricting the analysis to the E7-specific immunogens, the efficacy of Nef^mut^-engineered EVs strategy was 2 to 3-fold higher even than vaccines already in clinical trials.

The inherent variability of the methods used to evaluate anti-HPV immunity could affect the overall accuracy of the benchmark. In any case, the preclinical data we obtained in terms of anti-HPV/E7 CTL immunity seem to be more than promising in view of a possible translation into the clinic.

## 9. Conclusions

The characteristics of the Nef^mut^ CTL vaccine platform, together with the demonstrated flexibility in terms of incorporation of foreign antigens and ease of production, make the production of endogenously engineered EVs a powerful approach for novel antitumor immunotherapies, particularly anti-HPV tumors. Ongoing investigations in vivo on biodistribution and pharmacokinetic of engineered EVs will help to clarify the mechanisms underlying the observed induction of CTL immunity as well as the tissue districts involved. The introduction of combination therapies and monoclonal antibodies targeting the immune checkpoints blockade in cancer therapy has provided not only new opportunities for clinical success of already started experimentation of HPV therapeutic vaccines, but also new impulse for newly proposed therapeutic vaccines proven to be effective only in preclinical studies.

## Figures and Tables

**Table 1:** Anti-human papillomavirus (HPV) therapeutic vaccines in clinical trials [31].

Denomination	Description/Antigen	Adjuvant	Additional Treatment	Administration	Trial Design
TA-HPV [39]	Vaccinia Virus expressing E6 and E7 of HPV16 and 18		surgical procedure radiation therapy	i.m. injection	Phase II in patients with early CC
TVGV-1 GPI-0100 Placebo [40]	HPV16E7-PE (*Pseudomonas* exotoxin A); KDEL (ER retention signal) fusion protein	GPI-0100 (triterpene glycoside derived from saponins)		i.m. injection	Phase II randomized in double-blind patients with confirmed HPV-induced cervical HSIL
HspE7/ Poly-ICLC [41]	HSP65 of *Mycobacterium bovis* and E7 HPV16 fusion protein	Poly-ICLC/synthetic complex of carboxy-methylcellulose, polyinosinic-polycytidylic acid, and poly-L-lysine double-stranded RNA		i.m. injection	Phase I/II in patients with CIN III
Vvax001 [42]	Semliki Forest Virus vector encoding HPV-derived tumor antigens	Irradiated viral particles		i.m. injection	Phase I in patients with CIN 2, CIN 3, and CC
INO-3112 (VGX3100 INO-9012) [43,44,45]	DNA plasmids expressing E6 and E7 of HPV16 and 18	IL-12	Cisplatin; Radiotherapy; with or without Durvalumab (anti- PD-L1 mAb)	i.m. electroporation	Phase I/II in patients with CC, Head and Neck cancer; uterine cervical neoplasms
ISA101/ ISA101b [46]	13 overlapping 25-35-mer peptides from HPV16 E6 and E7 proteins	Pegylated IFN-γ	Carboplatin and paclitaxel; bevacizumab (anti VEGF-A mAb)	i.m. injection	Phase I/II in patients with Advanced or Recurrent HPV16 CC
DPX-E7 vaccine [47]	HPV16 E7-specific CTL peptide delivered by proprietary liposome formulation			i.m. injection	Phase I/II in HLA-A02 patients with head and Neck cancer, CC, Cancer of anus
PepCan [48,49]	4 HPV16-E6 peptides escalation doses	Candida albicans extract (Candin^®^)		i.d. injection	Phase I/II in women with HSIL
ADXS11-001 [50]	HPV 16 E7 fused to non-hemolytic listeriolysin O protein			i.m. injection	Phase II in patients with persistent, recurrent SCC or non-SCC
VB10.16 vaccine [51]	DNA expressing HPV16 E6-E7, a dimerization domain and an APC targeting domain			needle-free injection	Phase I/II in patients with CIN 2
GX-188E [52] Placebo	DNA expressing the E6/E7 fusion protein of HPV16 and 18, plus Flt3L and tPA sequences signals		Pembrolizumab (anti-PD1 mAb)	i.m. electroporation	Phase II randomized, double-blind, multi-center in patients with CIN II and CIN III
NGVL4a-Sig/E7(detox)/HSP70 [53]	Vaccinia virus expressing E6/E7; DNA plasmid encoding signal peptide, a detox form of HPV-16 E7 and the HSP70		Imiquimod	i.m. injection	Phase I in patients with HPV- precancerous lesions and CC
GX-188E GX-I7 [54]	DNA E6/E7 fusion proteins of HPV16 and 18 plus GX-I7	GX-I7 (IL-7 and hybrid Fc)	Imiquimod	i.m. electroporation	Phase 1 in patients HPV-positive
pNGVL4a-CRT/E7-Detox DNA Vaccine [55]	DNA HPV16 E7detox linked to calreticulin (CRT)		Cyclophosphamide intravenously up to 24 h	i.m. electroporation	Phase I in patients with Head and Neck Cancer
Ad-E6E7 MG1-E6E7 [56,57]	Adenovirus expressing E6 and E7 plus Oncolytic Maraba virus expressing E6 and E7		Atezolizumab (anti-PD-L1 mAb)	i.m.	Phase I

Abbreviations: CC (cervical cancer); HSIL (high grade intraepithelial cervical lesion); PE (*Pseudomonas aeruginosa* exotoxin A); CIN II and CIN III (cervical intraepithelial neoplasia of grade II and III); SCC (squamous cell carcinoma), APC (antigen presenting cell), Flt3L (Fms-like tyrosine kinase-3 ligand), tPA (tissue-type plasminogen activator), HSP (heat shock protein); CTL (cytotoxic T lymphocyte), i.m. (intra muscular), i.d. (intra dermal) injection.

**Table 2:** Comparison between the Nef^mut^ and the wild type Nef functions.

Nef Function	Nef^mut^ [68,69]	Wild-Type(wt)-Nef [70,71,72,73,74,75]
CD4 down-regulation	−	+++
Increase of HIV-1 (Human Immunodeficiency Virus 1) infectivity	−	+++
Class I MHC (Major Histocompatibility Complex) down regulation	−	++
PAK (p21-activated kinase) activation	−	+++
NAK (NF-kappaB-activating kinase) activation	−	+++
Exosome association	+++	+/−

**Table 3:** Strengths of the Nef^mut^-based CTL vaccine platform.

Strengths of the Nef^mut^-Based CTL Vaccine Platform
In vivo, endogenously engineering of exosomes with high therapeutic efficacy.
Overcoming the pitfalls of ex vivo or in vitro exosome production and isolation approaches.
Specificity of the immune response for the antigen of interest, with low risk of an immunogenic response to endogenously engineered exosomes.
Advantages in terms of development, production, costs, and safety compared to other exosome-based approaches.
Impact of the data obtained from the breast cancer HER2/Neu model of primary carcinogenesis.

**Table 4:** Percentage of CTL activation associated with DNA-based HPV therapeutic vaccines in mice. EV: extracellular vesicle.

Description DNA Vaccine Approach	Antigen	Administration	% CD8^+^ Activation
Nef^mut^ EV anchoring protein to generate immunogenic EVs Nef^mut^-EVs	HPV16 E7	i.m. injection	1.38% [77]
Intracellular targeting by LAMP-1, HSP70, CRT, Herpes Simplex Virus (HSV) VP22 sorting signals to enhance Ag presentation by APC	HPV16 E6	i.d. injection gold particles by gene gun	3% [33]
Simultaneous vaccination with E6+E7 fused to CRT to enhance Ag presentation by APC CRT sorting	HPV16 E6+E7	i.d. injection gold particles by gene gun	0.7% E6 0.4% E7 [34]
codon optimized E6	HPV16 E6	i.d. injection gold particles by gene gun	0.77% [35]
E6/E7 consensus sequences	HPV18 E6/E7	electroporation	0.21% [36]
E6/E7 consensus sequences	HPV16 E6/E7	i.m. injection	0.50% [81]
E6/E7 consensus sequences	HPV6 and HPV11 E6/E7	electroporation	0.5% HPV6 0.9% HPV11 [37]
GX-188: Shuffled E6 and E7 fragments+Flt3L and tPA signals to promote trafficking and Ag presentation	HPV16 and HPV18 E6/E7	electroporation	0.08% [38]
